# Quality of Working Life, Psychosocial Factors, *Burnout* Syndrome and Emotional Intelligence

**DOI:** 10.3390/ijerph17249550

**Published:** 2020-12-20

**Authors:** Eloísa Guerrero-Barona, Mónica Guerrero-Molina, Andrés García-Gómez, Juan Manuel Moreno-Manso, María Elena García-Baamonde

**Affiliations:** Department of Psychology, Faculty of Education, University of Extremadura, 06006 Badajoz, Spain; eloisa@unex.es (E.G.-B.); agarcil9@unex.es (A.G.-G.); jmmanso@unex.es (J.M.M.-M.); mgarsan@unex.es (M.E.G.-B.)

**Keywords:** quality of working life, burnout syndrome, emotional intelligence, psychosomatic symptoms, social support, conciliation of work and family life, satisfaction at work, professionals attending persons with intellectual disabilities

## Abstract

The objective of this work is to study the quality of working life associated to psychosocial factors and risks, *burnout* syndrome and emotional intelligence, as well as being able to detect predictors of the said syndrome. The sample consisted of 311 professionals working in direct contact with an intellectual disability from 15 associations of Extremadura (Spain). The Spanish version of the CESQT questionnaire was administered to evaluate burnout syndrome, the Wong & Law Emotional Intelligence Scale (WLEIS) was used to evaluate emotional intelligence, while the UNIPSICO Battery was used to evaluate the psychosocial factors of demands (work–family conflict and psychosomatic problems) and resources, such as social support and work satisfaction. The results indicate average values of burnout, revealing that work satisfaction, emotional intelligence, and social support are related to burnout syndrome. In addition, there are also positive correlations between psychosomatic symptoms and work–family conflict. Satisfaction at work, social support, and emotional intelligence (intrapersonal and interpersonal perception, use and regulation of emotions) predict burnout syndrome. What is more, the psychosomatic symptoms and work–family conflict explain, respectively, 17% and 17.9% of their variance. Thus, there is a need to develop intervention programs that encourage social support and the conciliation of family life, as well as training skills related to emotional intelligence, such as communication and conflict resolution.

## 1. Introduction

The quality of working life is a movement that appeared in the 1970 s in the USA with the aim of humanizing the work environment, paying special attention to the development of the human factor and improving the quality of people’s lives [1]. There are two theoretical–methodological perspectives which have dealt with the quality of life in the work environment and the quality of psychological life [2,3]. The latter perspective aims for the worker’s satisfaction, health and wellbeing, putting individual interests before those of the organization [4], while the former is oriented more towards achieving greater productivity and professional efficiency, as an initial step towards satisfying the needs and demands of each worker.

In accordance with the World Health Organization (WHO), economically active people spend a third of their time in the workplace where the conditions have considerable effects on their health [5]. Occupational health psychology studies the psychosocial factors at work and the quality of working life. It involves the application of the psychological principles to improve the quality of working life, the protection and promotion of workers’ safety, health, and wellbeing [6].

The psychosocial factors are conceived as the conditions present in work situations related to the organization of work, the environment, the type of position, and the tasks to be carried out, which all affect the development of both work and health. These factors can facilitate the activity, the quality of working life, and personal development, or, on the contrary, they can be an obstacle and interfere negatively, damaging both health and wellbeing at work. When this occurs, some authors have referred to them as psychosocial risk factors, as they have the potential to damage workers’ health [7].

The IV Survey on Work Conditions in Spain (Encuesta sobre Condiciones de Trabajo en España) carried out by the National Institute for Safety and Hygiene at Work (INSHT) demonstrated that psychosocial risks were one of the main causes of labor accidents and illness. Two reports from the European Agency for Safety and Health at Work in 31 European countries stressed the ever-increasing importance of emerging risks, considering them a problem for developing effective policies [8]. At the same time, such international organizations as the WHO have stated that some psychosocial risk factors, such as stress, burnout syndrome, or violence at work, are acquiring ever more relevance in developed countries [5].

Burnout syndrome has been conceptualized in different ways, depending on which of the two perspectives it has been considered. One, the clinical perspective, is oriented towards the diagnosis and understanding of SB as a state and effect of stress at work; while the other, which is of a psychosocial nature, is oriented towards explaining the development process and its trajectory, the result of the interaction between personal characteristics and the work context. This interactional and psychosocial perspective is the one we assume and the one that is the basis of this present work, as it is more explicative than diagnostic. To be precise, burnout syndrome is, from a psychosocial perspective, a response to chronic stress at work. It is a tridimensional syndrome that mainly affects professionals in support or service organizations and is characterized by high levels of emotional fatigue, depersonalization and low levels of personal fulfillment at work [9]. However, other works of research [7,10,11] have proposed that another component could be included: a sense of guilt. Although it would not be present in all professionals, when present, it would give rise to two profiles and patterns of the development of the syndrome; one related to work stress that causes a moderate, but not incapacitating malaise, and a second, more seriously limiting profile that interferes in the subject’s ability to function both at work and outside work, incapacitating him or her [12]. Thus, the repercussions concerning health are clear, as they can directly affect one’s health through psychosomatic symptoms [13], or they may be expressed as a blockage of one’s emotional expression [14].

There have been several works of research that have shown that professionals who work with persons who have an intellectual disability are subjected to numerous psychosocial factors and high levels of stress [15]. Work overload and a lack of support from colleagues [15,16], no participation in the decision-taking, the user’s disability [17], family-work conflicts and the emotional demands or conflicts of the role [18] are all factors that explain the occurrence of burnout in this population.

On the other hand, there are very few works that have analyzed the protective factors for the health and wellbeing of professionals who work with persons who have a disability, since the majority of studies concerning this population have focused on the prevalence of burnout syndrome [16]. Some works have also demonstrated the role played by the quality of one’s working life in the wellbeing of the worker as well as in the quality of the service being given [19]. Along these lines, several psychosocial factors, such as satisfaction at work [20], family life-work conciliation and interpersonal relations [4], or emotional intelligence [21], have all been related to burnout syndrome. In particular, in a review carried out by Pérez-Zapata & Zurita [20], it has been stated that satisfaction at work was the principal construct influencing the general results concerning the quality of one’s working life.

Satisfaction at work refers to the positive feeling that employees have about their jobs [22]. There is sufficient scientific evidence concerning the influence it can have on burnout and vice versa. The studies that have examined the said relationship conclude that both constructs maintain a negative relationship one with the other [23,24,25,26]. Although there is a certain amount of controversy about which construct predicts and which is predicted, both transversal and longitudinal studies have carried out research which suggests that satisfaction at work is a predictor of burnout, indicating that high levels of satisfaction at work may diminish burnout syndrome in workers [27,28].

Other works of research [29] have considered that interpersonal relations may become a source of stress; however, when there is good interpersonal communication and perceived social support, then the negative effects of stress on health can be lessened.

Another related variable that has been studied is emotional intelligence, conceived as an ability to receive, assimilate, understand and regulate one’s own emotions and those of others [30]. In accordance with Extremera, Durán, and Rey [19], emotional intelligence can favor the management and repair of the emotions that arise in the work context, as well as an understanding of social situations at the workplace of these professionals. In addition, some works of research have shown that those workers who have a high emotional intelligence tend to be more satisfied in their sphere of work [31,32,33], while also decreasing the negative effects of the so-called “core factors of burnout”: emotional exhaustion and cynicism [34,35].

The Dual Spiral Model [36,37], part of the Model of the Dual Process of Schaufeli and Bakker [38], combines the traditional, more negative approach to stress at work with a more positive approach. It therefore grants special relevance to the study of the positive results of the work, such as satisfaction at work, and not only the negative consequences of stress, such as burnout. In this sense, the model includes personal resources and concludes that these not only serve to provide balance to the demands, but also provide a motivational potential. Thus, stress and the experience of positive consequences at work can be explained with respect to both the work and personal demands and resources [39].

As for the personal variables, a review carried out by Gil-Monte [40] demonstrated that, of the personality variables related to burnout, a resistant personality, locus of control, and type A behavior pattern stand out. In addition, considering the personality dimensions of the big five model, some works of research have stated that neuroticism correlates with burnout [41,42,43]. One of the results of this last study showed that neuroticism was the only variable that predicted emotional fatigue. More recently, significant relations have been found between extraversion and depersonalization [44] and positive correlations between certain dimensions of burnout, depression, and the personality factors of neuroticism, kindness, responsibility, extraversion, and anxiety [45].

Although they are far from the objectives of this work, some other variables and personal resources, which have been analyzed in more recent works, such as self-efficacy [46,47,48,49], engagement [17,50], satisfaction with life [51], and prosocial motivation have been included as variables that can lessen the deterioration in relations between the person with a disability and their carer [52]. We also include resilience, as burnout syndrome is related to low levels of self-efficacy and resilient behavior [47]. Other research works have stated that self-determined motivation is positively related to positive affection and negatively to negative affection and to burnout syndrome [53].

In this work, we use indistinctly psychosocial risk factors and demand factors. It is also necessary to make it clear that we use the term resource or protection factors to refer to those factors that protect health and wellbeing, such as satisfaction at work and social support.

Using the Dual-Spiral Model of Salanova, Bresó, and Schaufeli [36] and Schaufeli and Bakker [38] as the basis of our work, while also considering previous studies, we aim to study the possible relation between the quality of one’s working life associated to the psychosocial factors of resources and demands, burnout syndrome, and emotional intelligence, as well as detecting predictors of the said syndrome in professionals working in direct contact with persons with an intellectual disability. To be precise, we believe that professionals suffering from burnout syndrome will show a lower emotional intelligence, work satisfaction and social support (Hypothesis 1); and that both the factors of psychosocial demands and resources and emotional intelligence will allow us to predict burnout syndrome (Hypothesis 2).

## 2. Method

### 2.1. Sample

The participants were selected using a non-probabilistic convenience sample. The sample consists of 331 professionals from ‘Plena Inclusión Extremadura’ (Full inclusion Extremadura) who work in contact with persons with a intellectual disability from 15 different associations in Extremadura (Spain), between 22 and 63 years of age (*M* = 37.98; *DT* = 10.30). Women represent 72.1% of the workers surveyed, and of the workers, while 79% have a stable partner and 56.7% have dependent children.

As for the different working positions analyzed, there is a predominance of careers education, who make up 36% of those interviewed, followed by 13.5% who are teachers. The majority (58.2%) have permanent contracts, while 50% had been in their work positions for under 7 years. As for the services in which they carried out their duties, 22.2% did so in Day Centers, el 20.52% in Occupational Centers, 19.22% in Residences and 16.61% in Special Education Centers.

### 2.2. Instruments

The Questionnaire to Evaluate the Burnout Syndrome at Work (CESQT) by Gil-Monte [12], was designed to evaluate burnout syndrome through measurements of a person’s cognition, emotions and attitudes concerning their work experiences [12]. It is made up of 20 items that use a frequency response format of five points, from Never (0) to Very frequently: every day (4). In this case, we use the version which estimates the variables mentioned above through four scales: work engagement (5 items), mental exhaustion (4 items), apathy (6 items), and guilt (5 items). Work engagement measures the individual’s desire to reach the job’s goals as this is supposedly a source of personal satisfaction; mental exhaustion is the appearance of emotional and physical exhaustion due to having daily contact at work with persons who have or cause problems; apathy is defined as the presence of negative attitudes of indifference and cynicism towards the organization’s clients; while guilt is defined as the appearance of feelings of guilt due to the negative behavior and attitudes at work, in particular towards those persons with whom they establish work relationships. The scales of work engagement, mental exhaustion, and apathy allow us to obtain a general *burnout* score. With the exception of the dimension work engagement, high scores indicate high levels of *burnout*. Previous studies by the same author obtained reliability indices of α = 0.77 for engagement, α = 0.86 for exhaustion, α = 0.75 for apathy and α = 0.79 for guilt.

The UNIPSICO Battery of Gil-Monte [54] allows us to evaluate the psychosocial situation of the workers. This instrument is flexible and can include different validated scales. For our study, we used the scales that evaluate the psychosocial factors of resources, measuring the social support (6 items); the consequences of the psychosocial risks, measuring job satisfaction (6 items) and psychosomatic problems (9 items); and it is completed by adding a scale that evaluates work–family conflict (6 items). High scores in the scales that evaluate demands indicate the presence of psychosocial risks; while, in the case of resources, the indices of psychosocial risk are determined by low scores.

The Wong & Law Emotional Intelligence Scale (WLEIS) by Wong and Law [55] allows us to measure the Emotional Intelligence (EI). The aim of this is to evaluate the level of perceived EI and consists of 16 items scored on a Likert type scale of 7 points, where 0 is Never, 1 = Almost never, 2 = Rarely, 3 = Sometimes, 4 = Often, 5 = Frequently and 6 is Always. In addition, the scale allows four dimensions of the EI to be evaluated: intrapersonal perception or the evaluation of one’s own emotions; interpersonal perception or the evaluation of the emotions of others; use of the emotions or assimilation; and regulation of the emotions. The reliability index for the subscales is of α = 0.87 for intrapersonal, α = 0.90 for interpersonal, α = 0.84 for use of emotions and α = 0.83 for regulation of emotions.

The choice of these instruments is based not only on the objectives of this study, but also on the goodness of the psychometric characteristics of all of them. In addition, we chose not to use adaptations of English language questionnaires, as there exist instruments in Spain that allow us to evaluate the constructs being studied which present adequate technical characteristics of psychometric validity.

### 2.3. Procedure

We first contacted ‘Plena Inclusión de Extremadura’ to inform the director of the objectives and phases of the research. We requested his collaboration in spreading the word in order to make those in charge of the 15 associations aware of our study, and thus help to bring it to the attention of the professionals in each association, since their participation in the study was of a voluntary nature.

Thus, after having been informed by the research team, a technician specialized in applied psycho-sociology and trained in labor risk prevention from Semfex, part of ‘Plena Inclusión Extremadura’, interviewed the directors of the 15 associations to inform them of the objectives and different phases of the research. They were all asked to collaborate and to inform and encourage the workers of each association to participate in the study. Those in charge of the associations promised to call an informative meeting for all the professionals of the association and to facilitate a list of those willing to participate.

Finally, having received the list of participants, the risk prevention technician set up appointments with all the associations to meet the participants and explain to them the objectives of the study. They were informed that they could complete the questionnaire during their work hours following authorization from their superiors and that it would take approximately 15 min. They were also informed of the procedure for collecting the questionnaires and the deadlines for submission. The anonymity of their answers was assured. The data collection process lasted a month and a half.

All procedures performed in studies involving human participants were in accordance with the ethical standards of the institutional research committee. Ethical clearance was obtained from the Bioethics and Biosecurity Commission of University of Extremadura (Ref. 185/2020).

### 2.4. Statistical Treatment

The statistical analysis of the data was carried out using the IBM SPSS Statistics v. 25.0. (IBM, Armonk, NY, USA). We first carried out a descriptive analysis of each of the variables included in the study (burnout syndrome, resource and demand, psychosocial factors, and emotional intelligence). Then, attending to the sample size, we carried out a correlation analysis using Pearson’s coefficient to analyze the relation between burnout syndrome, the psychosocial factors, and emotional intelligence. Finally, we performed a linear regression analysis to determine to what extent the psychosocial factors and emotional intelligence can significantly predict burnout syndrome among the workers.

## 3. Results

Attending to the criteria offered for the interpretation of the CESQT, for all the dimensions, as well as for the general scale, the sample presents average values, although it is the women over 42 years of age who show the highest average scores (*M* = 0.78; *SD* = 0.58). The highest score is in the dimension of *engagement* (*M* = 3.39; *SD* = 0.72), in which that of men aged 22 to 42 stand out (*M* = 3.53; *DT* = 0.64); while the lowest scores are in the subscale apathy (*M* = 0.5; *SD* = 0.49), so the sample does not show high levels for burnout syndrome (Table 1).

As for the scores of the battery evaluating the psychosocial factors of resources and risks, the participants obtained high scores in the dimension of psychosocial resources social support (*M* = 2.64; *SD* = 0.93), and in satisfaction at work (*M* = 2.43; *SD* = 0.67), although it was women over 42 years of age who presented the lowest average scores (*M* = 2.29; *SD* = 0.64). Furthermore, the lowest average score among the participants was to be found in the dimension psychosomatic problems (*M* = 0.64; *SD* = 0.61), with higher scores among the women over 42 years of age (*M* = 0.73; *SD* = 0.65).

As for emotional intelligence, the results obtained in the general WLEIS scale show average scores in emotional intelligence (*M* = 3.20; *SD* = 0.43), in which those of men over 42 years of age stand out (*M* = 3.22; *SD* = 0.40). The highest score appears in the subscale of intrapersonal perception (*M* = 3.36; *SD* = 0.49), which evaluates a person’s own emotions, while the lowest score is to be found in regulating the emotions (*M* = 3.07; *SD* = 0.61).

As for the hypothesis which postulates that those professionals suffering from burnout syndrome have a lower emotional intelligence, job satisfaction and social support; the following data can be extracted from the correlation analysis of the variables, as reflected in Table 2.

Age correlates with guilt (*r* = 0.207, *p* = 0.001) and job satisfaction (*r* = −0.122, *p* = 0.036), in such a way that as workers get older, they have an increased sense of guilt and lower job satisfaction.

The number of dependent children the participants have negatively correlates with job satisfaction (*r* = −0.212, *p* = 0.002) and social support (*r* = −0.166, *p* = 0.017), so we can state that as the number of dependent children increases, both job satisfaction and social support decrease.

As for the number of years in workplace, as the number of years of the participants’ work experience increases, so do the feelings of guilt (*r* = 0.124, *p* = 0.048).

In both the general CESQT scale and the dimension exhaustion, we obtain significant correlations with the rest of the variables included in the study. In this sense, it must be pointed out that, with respect to burnout syndrome, there are positive correlations in the cases of psychosomatic symptoms (*r* = 0.412, *p* < 0.001) and work–family conflict (*r* = 0.423, *p* < 0.001), while it is related negatively to job satisfaction (*r* = −0.324, *p* < 0.001), social support (*r* = −.247, *p* < 0.001), and emotional intelligence (*r* = −0.320, *p* < 0.001).

Both the general WLEIS scale and the subscales use of emotions and regulating emotions are related to the rest of the variables under study. To be precise, emotional intelligence correlates negatively with the psychosocial dimensions psychosomatic symptoms (*r* = −0.203, *p* < 0.001) and work–family conflict (*r* = −0.270, *p* < 0.001). It can thus be said that, as emotional intelligence increases in the participants, the psychosomatic symptoms and work–family conflict decrease. Similarly, it is also worth noting that there is a positive correlation present between emotional intelligence and job satisfaction (*r* = 0.220, *p* < 0.001) and social support (*r* = 0.268, *p* = 0.004), so a high level of emotional intelligence is related to high scores in job satisfaction and social support.

Finally, with respect to the rest of the subscales, intrapersonal perception correlates with the general scale of the *burnout* syndrome (*r* = −0.213, *p* = 0.001), with the subscales of the CESQT exhaustion (*r* = −0.254, *p* < 0.001), apathy (*r* = −0.155, *p* = 0.009) and guilt (*r* = −0.255, *p* < 0.001), and the subscales of the UNIPSICO psychosomatic symptoms (*r* = −0.159, *p* = 0.006) and work–family conflict (*r* = −0.238, *p* < 0.001). Similarly, interpersonal perception is related to the general scale of the CESQT (*r* = −0.247, *p* < 0.001) and its subscales *engagement* (*r* = 0.168, *p* = 0.004), exhaustion (*r* = −0.183, *p* = 0.001), apathy (*r* = −0.146, *p* = 0.014) and guilt (*r* = −0.215, *p* < 0.001), as well as with the subscales work–family conflict (*r* = −0.179, *p* = 0.002), satisfaction (*r* = 0.161, *p* = 0.005) and social support (*r* = −0.162, *p* = 0.005). The results allow us to see that the greater the emotional intelligence, the lower the scores will be in burnout and psychosocial risks.

Furthermore, the analysis of the results of the correlations shows us that there is a justification for controlling the participant’s age, number of dependent children and number of years working in our hypotheses.

Table 3 shows the results obtained with respect to the hypothesis which states that both psychosocial factors and emotional intelligence allow us to predict burnout syndrome controlling the effect of the participants’ age and gender.

The data indicate (Table 3) that job satisfaction explains 11.2% of the variability determined in the scores concerning burnout syndrome (β = −0.330; *p* < 0.001) and 5.8% (β = −0.232; *p* < 0.001) in those concerning social support. Similarly, psychosomatic symptoms explain 16.6% of the variance in the responses concerning *burnout* (β = 0.406; *p* < 0.001), and 18.3% (β = 0.425; *p* < 0.001) of those concerning work–family conflict. Thus, 31.1% of the variance is explained by the set of psychosocial factors.

With respect to the general scale of emotional intelligence (β = −.319; *p* < 0.001) and its dimensions intrapersonal perception (β = −0.212; *p* = 0.001), interpersonal perception (β = −0.249; *p* = 0.001), use of emotions (β = −0.313; *p* < 0.001) and regulation of emotions (β = −0.257; *p* < 0.001), these allow us to predict burnout syndrome. In this sense, we can confirm that 11.9% of the variance in burnout syndrome is explained by emotional intelligence.

## 4. Discussion

In this study, our proposed objective was to study the possible relationship between the quality of one’s working life to the resource and demand psychosocial factors, burnout syndrome, and emotional intelligence; as well as to detect predictors of the said syndrome in professionals working in direct contact with persons who have an intellectual disability.

The results indicate that such professionals do not have high levels of burnout, show a high level of job satisfaction and social support, and adequate emotional intelligence. These results are in line with previous studies [56,57]. As the authors set out, it can be confirmed that the social system of the organizations is a highly relevant context for a person’s workplace health. However, the results diverge from those set out by Arias et al. [58] and González and Veliz [59], since they stressed that, of all the professional groups evaluated, that of carers of persons with a disability was the group that showed the greatest psychosocial risk due to the high percentage of subjects suffering from burnout syndrome.

Based on the results of this research, we can confirm the hypothesis that professionals suffering from burnout show lower emotional intelligence, job satisfaction and social support. To be precise, with respect to the relationship between burnout syndrome and the resources and demands psychosocial factors, our results are on the same lines as those of Pérez-Zapata and Zurita [20]. Burnout syndrome is related to psychosomatic symptoms, work–family conflict, job satisfaction, and social support. In this sense, the presence of psychosomatic symptoms and work–family conflicts, as well as lower job satisfaction and social support are all associated with the existence of burnout syndrome among professionals working with persons who have an intellectual disability, in agreement with Gil-Monte [60] and Lazarus and Folkman [61]. The factors of work demand and the presence of psychosomatic symptoms are indicators of a low quality of workplace life. On the other hand, these relations demonstrate the weight, repercussions and importance that resources psychosocial factors have with respect to the quality of one’s working life. To be more precise, the results show that a lack of social support in an organization may favor the appearance of burnout syndrome [62].

Similarly, in intrapersonal perception, interpersonal perception, use of emotions and regulation of emotions. Thus, in line with Extremera, Durán, and Rey [19] and Pena and Extremera [63], we can confirm that emotional intelligence is a factor that protects against the appearance of burnout, since it can repair the tensions that arise in the work context. Thus, a good use of these emotional skills can lead to a greater control over the stressors to be found in the workplace [64]. Moreover, emotional intelligence is related to job satisfaction [50,65,66,67,68] and social support [69], thus diminishing the load of the psychosocial demand factors [34,35].

In general terms, we can confirm that burnout is related to the rest of the variables included in this study, which reveals the importance of preventing this syndrome in order to encourage an adequate quality of life in the workplace.

As for the second hypothesis, which poses the idea that both the psychosocial factors of resources and demands and emotional intelligence will allow us to predict burnout, we can confirm the said prediction of burnout through the psychosocial factors and emotional intelligence. Going into the results obtained in greater depth, we can conclude that while the psychosomatic symptoms and the existence of work–family conflicts can predict burnout, job satisfaction, and social support in the workplace can predict a lower presence of burnout among those professionals who attend to persons with an intellectual disability. Furthermore, lower scores in emotional intelligence also allow us to predict the presence of burnout. These conclusions coincide with the works of Durán, Extremera, and Rey [70] and Veliz et al. [71], which confirm that emotional intelligence and wellbeing at work can function as protective factors against stress.

Thus, in view of the work set out in Salanova et al. [39], the results obtained allow us to study the psychosocial health of those professionals using an approach that includes both wellbeing and quality of life in the workplace. The high demands loaded upon the employees, which may lead to burnout, as well as a decline in health and increased absenteeism. Nevertheless, the availability of resources can stimulate such positive results at work as commitment to the job and a reduction in absenteeism and burnout.

The study of the quality of life in the workplace is approached from two theoretical-methodological perspectives: the quality of life in the workplace and the perspective of the psychological quality of life. In short, it is a question of reconciling the objectives of the individuals with those of the organization [72]. This means it is essential to provide the professionals with the skills and competences necessary for them to be able to act as protective factors, as well as in the aspects of the organization that influence the quality of life at work of the population under study.

The relationship between burnout syndrome and job satisfaction is fully explained, so we can undertake lines of action aimed at increasing the levels of job satisfaction as a preventive measure against the appearance of burnout. This leads to intervening in those aspects that encourage work engagement which, according to the model of two factors of Helzberg, Mausner, and Snyderman [73], would be related to carrying out stimulating work tasks, a feeling of self-fulfillment, recognition, achievements and responsibility.

On the other hand, the psychosomatic symptoms are objective indicators of a low quality of life in the workplace, and the seriousness of the problems can lead to inadequate responses that hinder the relief of both physical and emotional symptoms. In this sense, workers should be trained in effective strategies to face problems at work, as well as establishing tools and protocols for effectively resolving conflicts [46]. However, work–family conflict is the risk factor that appears most in this study. This fact may be due to the existence of a larger proportion of female workers and the demands of home life and work that they face. This leads us to the necessity of establishing conciliation measures in order to avoid the problems related to the quality of life at work and home [74,75].

According to Extremera, Rey, and Pena [76], emotional intelligence acts as a protective factor against the appearance of stress. However, its influence with respect to job performance is greater because, in a job market such as that which exists at this moment, there is an ever-increasing demand for persons competent in such aspects as communication, social skills, and decision-taking, and such skills are ever more highly valued. Thus, it is vital for persons to be trained in all the areas related to emotional intelligence [77] since, as pointed out by Salovey, Mayer, and Caruso [21], emotional intelligence will influence the development of positive states and moods in both the personal sphere and that related to work experience. This is so because the emotions play a crucial role in how people adapt to the psychosocial risks in the workplace in socio-community contexts [52], which in turn may explain the quality of service presented and the client’s satisfaction [78].

Finally, our study reveals the need for interventions in the aspect of social support; more precisely, social support perceived from the point of view of management, though without discarding the importance of continually encouraging social support among fellow workers or from the perspective of supervisors. It is currently recommended to carry out periodic evaluations of the psychosocial risks present in the workplace and their consequences, to be able to modify those that lead to the appearance of burnout, improve the communication network, and promote the participation of the organization [42].

Before we finish, we feel it is necessary to mention some of the limitations of this research. One is related to the selection, size and homogeneity of the sample. The selection of the participants was carried out using a non-probabilistic convenience sampling. Given the characteristics of the collective and the institutional facilities and support we received to gain access to the 15 associations, sampling techniques were not applied.

As for the size of the sample, there were 4 associations that did not participate for unknown reasons not connected with this study. In addition, 9 questionnaires had to be discarded as they were incomplete. Both circumstances caused an important loss of information. These reasons may explain why the sample size was lower than expected.

In addition, on using a transversal design, the data obtained do not allow the temporal evolution of the scores to be followed, nor do they allow causal relations to be established between the variables included in this study. Similarly, relationships have been noted between the psychosocial factors of resources and burnout syndrome with the professional’s age, number of years of experience in the job and number of dependent children. Thus, in future lines of research, it would be desirable to include and control other variables and to carry out studies with longitudinal designs that would allow us to infer causal relations between burnout, risk factors and psychosocial protection and emotional intelligence.

On the other hand, although the indices of internal consistency found in the study are acceptable in most scales used, it should be pointed out that the reliability of the subscale apathy is questionable, since the analysis points to a low correlation between the items, so we should be cautious on interpreting these particular results.

## 5. Conclusions

In short, our results invite reflection concerning the practical relevance of these data and how they impact the global working of the organization. Therefore, we find ourselves under the obligation to plan intervention programs that can contribute to improving the quality of life in the workplace for the workers, the quality of the services provided and, consequently, the quality of life and wellbeing of those persons with an intellectual disability.

We are convinced that the aim of the research must not only be the evaluation and management of the psychosocial risks, but also the application and development of policies of good organizational practices to avoid the appearance of burnout and the costs it brings with it. Any intervention must be supported by a theoretical foundation and scientific evidence. We hope that this study contributes to realizing the next intervention aimed at the collective of professionals who attend to persons with an intellectual disability will be sustained by practices based upon evidence. As suggested more than a decade ago by two of the great references in the study of burnout syndrome, Gil-Monte [40] and Moreno and Garragosa [79], the interventions must comply with a series of requirements: they must be systematic and accessible to all the professionals of the organization and they must be based on problem solving with a well-defined plan of objectives. Furthermore, they must be focused on formation and training on three levels: organizational (programs of anticipatory socialization, feedback programs for the professionals concerning their performance and organizational development through diagnostics, evaluation, and intervention), interpersonal (social support and training in social skills), and individual (emotional intelligence). Whatever the prevention or intervention program implemented, it must include an informative module explaining what burnout syndrome is and it must also encourage the development of professional and personal skills and competences.

## Figures and Tables

**Table 1:** Shows the descriptive analysis of the studied variables. Means, standard deviations, and centiles of the instruments.

	Males (22 to 42 Years)		Males (Over 42 Years)		Females (22 to 42 Years)		Females (Over 42 Years)						
Scale	*M*	*SD*	*M*	*SD*	*M*	*SD*	*M*	*SD*	*M*	*SD*	Range	*P*	α
**CESQT**	0.67	0.48	0.75	0.50	0.73	0.43	0.78	0.58	0.77	0.48	0–4	40	0.77
Engagement	3.53	0.64	3.32	0.69	3.41	0.68	3.34	0.87	3.39	0.72	0–4	50–55	0.82
Exhaustion	1.24	0.87	1.39	0.85	1.38	0.82	1.42	0.85	1.38	0.83	0–4	45–50	0.81
Apathy	0.53	0.57	0.48	0.47	0.49	0.49	0.47	0.44	0.5	0.49	0–4	35	0.59
Guilt	0.67	0.57	0.99	0.76	0.57	0.55	0.70	0.57	0.66	0.59	0–4	45	0.72
**UNIPSICO**													
Social support	2.70	0.92	2.70	0.94	2.69	0.92	2.48	0.89	2.64	0.93	0–4		0.83
Job satisfaction	2.55	0.65	2.46	0.59	2.46	0.69	2.29	0.64	2.43	0.67	0–4		0.73
Psychosomatic problems	0.47	0.39	0.51	0.39	0.66	0.65	0.73	0.65	0.64	0.61	0–4		0.90
Work–family conflict	0.59	0.59	1.00	0.98	0.79	0.64	0.61	0.52	0.74	0.66	0–4		0.75
**WLEIS**	3.22	0.40	3.23	0.31	3.20	0.46	3.22	0.46	3.20	0.43	0–4		0.89
Intrapersonal perception	3.37	0.49	3.42	0.43	3.34	0.51	3.38	0.45	3.36	0.49	0–6		0.76
Interpersonal perception	3.11	0.44	3.13	0.45	3.18	0.47	3.23	0.43	3.17	0.45	0–6		0.68
Use of emotions	3.27	0.57	3.26	0.46	3.21	0.62	3.23	0.62	3.22	0.59	0–6		0.75
Regulation of emotions	3.15	0.55	3.11	0.54	3.06	0.61	3.03	0.66	3.07	0.61	0–6		0.83

**Table 2:** Correlation statistics.

	1	2	3	4	5	6	7	8	9	10	11	12	13	14	15	16	17
**1 Age**	--																
**2 Dependent children**	0.457 **	--															
**3 Years in the job**	0.659 **	0.316 **	--														
**4 CESQT**	0.022	0.022	−0.032	--													
**5 Engagement**	−0.041	−0.063	0.044	−0.761 **	--												
**6 Exhaustion**	0.030	0.020	−0.008	0.783 **	−0.377 **	--											
**7 Apathy**	−0.043	−0.028	−0.017	0.674 **	−0.250 **	0.365 **	--										
**8 Guilt**	0.207 **	0.044	0.124 *	0.226 **	−0.147 *	0.202**	0.242 **	--									
**9 Psychosomatic pr.**	0.014	0.068	−0.003	0.412 **	−0.325 **	0.533 **	0.133 *	0.104	--								
**10 Work–family conflict**	0.013	0.133	0.021	0.423 **	−0.251 **	0.436 **	0.241 **	0.118 *	0.375 **	--							
**11 Job satisfaction**	−0.122 *	−0.212 **	−0.086	−0.324 **	0.406 **	−0.264 **	−0.137 *	−0.073	−0.280 **	−0.207 **	--						
**12 Social support**	−0.084	−0.166 *	−0.012	−0.247 **	0.389 **	−0.204 **	0.010	0.050	−0.307 **	−0.298 *	0.451 **	--					
**13 WLEIS**	−0.027	−0.005	0.032	−0.320 **	0.203 **	−0.277 **	−0.209 **	−0.351 **	−0.203 **	−0.270 **	0.220 **	0.168 **	--				
**14 Intrapersonal perception**	0.013	0.035	0.049	−0.213 **	0.070	−0.254 **	−0.155 **	−0.255 **	−0.159 **	−0.238 **	0.092	0.088	0.841 **	--			
**15 Interpersonal perception**	0.018	−0.034	0.058	−0.247 **	0.168 **	−0.183 **	−0.146 *	−0.215 **	−0.030	−0.179 **	0.161**	0.162 **	0.733 **	0.520 **	--		
**16 Use of emotions**	−0.036	−0.007	0.003	−0.318 **	0.263 **	−0.286 **	−0.159 **	−0.346 **	−0.244 **	−0.250 **	0.182**	0.175 **	0.845 **	0.623 **	0.515 **	--	
**17 Regulation of emotions**	−0.067	−0.010	0.006	−0.255 **	0.142 *	−0.179 **	−0.212 **	−0.303 **	−0.192 **	−0.212 **	0.257 **	0.119 *	0.827 **	0.617 **	0.441 **	0.567 **	--

** *p* < 0.01 * *p* < 0.05.

**Table 3:** Linear regression analysis of the Questionnaire to Evaluate the Burnout Syndrome at Work (CESQT) with respect to the psychosocial factors and emotional intelligence.

CESQT				
	*r*	β	*t*	*p*
Psychosomatic problems	0.407	0.406	6.858	0.000
Work–family conflict	0.428	0.425	70.386	0.000
Job satisfaction	0.334	−0.330	−5.421	0.000
Social support	0.241	−0.232	−3.669	0.000
WLEIS	0.324	−0.319	−5.332	0.000
Intrapersonal perception	0.219	−0.212	−3.436	0.001
Interpersonal perception	0.254	−0.249	−4.055	0.000
Use of emotions	0.318	−0.313	−5.222	0.000
Regulating emotions	0.262	−0.257	−4.200	0.000
